# Clusterin/Apolipoprotein J immunolocalization on carotid artery is affected by TNF-alpha, cigarette smoking and anti-platelet treatment

**DOI:** 10.1186/1476-511X-13-70

**Published:** 2014-04-23

**Authors:** Amalia E Yanni, George Agrogiannis, Christos Gkekas, Despina Perrea

**Affiliations:** 1 Department of Nutrition and Dietetics, Harokopio University of Athens, 70 El Venizelou Ave, Athens, Greece; 2 Department of Pathology, Medical School, National and Kapodistrian University of Athens, Goudi, Athens, Greece; 3 Department of Vascular Surgery, Red Cross Hospital, 1 Athanasaki Str, Athens, Greece; 4 Laboratory of Experimental Surgery and Surgery Research, Medical School, National and Kapodistrian University of Athens, 15B Ag Thoma Str, Goudi, Athens, Greece

**Keywords:** Apolipoprotein J/Clusterin, Carotid artery disease, Cellular stress, Inflammation, Anti-platelet treatment, Tumor necrosis factor-a, Smoking

## Abstract

**Background:**

Clusterin (CLU) /Apolipoprotein J is a protein biosensor of oxidative stress and inflammation, which is upregulated in many pathological processes including atherosclerosis. Previous studies have shown that in aortic tissue, CLU expression increases with atherosclerotic lesion progression and it has been coupled with vascular damage and coronary artery disease. A few studies enter into CLU and carotid atherosclerosis while the apolipoprotein’s expression on human carotid tissue and its association with parameters related to the disease development has not been examined. The present study was designed to reveal the relationships between the degree of CLU immunolocalization on carotid artery and demographic characteristics, blood parameters and pharmacological treatment of patients underwent internal carotid artery endarterectomy.

**Methods:**

CLU expression was detected by immunohistochemistry in 42 carotid endarterectomy specimens. Patients’ serum levels of tumor necrosis factor-a (TNF-a), interleukin-6 (IL-6), high sensitive C-reactive protein (hsCRP) and classical parameters related to atherosclerosis such as lipid profile, as well as thrombosis related parameters such as fibrinogen, antithrombin III, protein C and protein S were determined. Demographic characteristics, smoking habits and the use of medications were recorded. Comparisons between groups were performed by students’*t*-test and analysis of variance. Independent associations with CLU expression on carotid tissue were denoted by linear regression analysis.

**Results:**

CLU imuunolocalization was denser in smokers than in non-smokers (*p* = 0.041) while it was rarefied in specimens of patients on cropidogrel treatment (*p* = 0.045) compared to the rest not taking this medication. Clopidogrel intake was independent predictor of lower CLU expression on carotid artery (*p* =0.045). CLU was positively correlated with serum TNF-a concentration (*r* = 0.33, *p* = 0.040) that was independent predictor of higher expression of the apolipoprotein (*p* = 0.001). IL-6, hsCRP and classical parameters related to atherosclerosis and thrombosis were not associated with CLU immunolocalization.

**Conclusion:**

Our study suggests that CLU expression on carotid artery is affected by TNF-alpha, cigarette smoking confirming its association with oxidative and cellular stress and anti-platelet medication reflecting the protective effects of such pharmacological treatment on vascular wall.

## Background

Clusterin (CLU)/Apolipoprotein J is a highly conserved glycoprotein which has attracted significant scientific attention since it is implicated in many biological processes and exerts a broad spectrum of functional properties. It is a heterodimeric protein with molecular weight of 75–80 kDa, which consists of two subunits fit together by five disulfidic bonds. CLU in humans is encoded by a single copy-gene, which is located on chromosome 8p21. The wide distribution in different tissues indicates the importance of its biological roles [1].

CLU is involved in lipid transport since it is associated with high-density lipoproteins (HDL) in plasma, specifically with particles containing apolipoprotein A-1 and cholesteryl ester transfer protein (CETP) [2]. It has been shown that induces cholesterol export from macrophage foam cells [3], transports lipids during cell differentiation and cell death [4] and stabilizes stressed proteins [5]. A number of studies presented that is implicated in aging and age-related diseases as neurodegeneration, diabetes and atherosclerosis [6-8] and acts as a biomarker of cellular senescence and oxidative stress [9].

The role of CLU in atherosclerosis remains largely unknown. Since cellular stress is engaged with the pathogenesis of the disease, the study of the apolipoprotein expression on vascular tissue and the elucidation of underlying mechanism merit scientific interest. Studies have shown that CLU distribution in human aorta is increased with the progression of atherosclerosis indicating a protective response to oxidative stress [7,10,11].

To our knowledge, a few studies enter into CLU and carotid atherosclerosis [12,13] while apolipoprotein’s expression on human carotid tissue and its association with parameters related to the disease development has not been examined.

The present study was designed aiming to investigate the possible association between CLU immunolocalization on carotid artery of patients underwent endarterectomy with demographic characteristics, pharmacological treatment and blood parameters (biochemical, hematological, thrombosis related parameters and inflammatory factors).

## Results

Study population characteristics are recorded on Table 1 while blood parameters and histological data are presented on Table 2.

**Table 1:** Study population characteristics

**Study population characteristics**	***n*** **= 42**
Age (years)	65.9 ± 7.7
Male gender (%)	76.2
BMI (kg/m^2^)	27.8 ± 4.1
Clinical symptomatology (%)	50.0
CAD (%)	30.0
Smoking (%)	71.4
Hypertension (%)	83.3
Type II diabetes mellitus (%)	31.0
*β*-Blockers (%)	32.5
ACE inhibitors (%)	37.5
CaCB (%)	45.0
Diuretics (%)	15.0
Clopidogrel (%)	50.0
ASA (%)	45.0
Antidiabetics (%)	25.0
Insulin (%)	5.0
Statins (%)	38.1

Values of age and BMI are expressed as mean ± SD. *BMI*, body mass index; *CAD*, coronary artery disease; *ACE,* angiotensin converting enzyme; *CaCB,* calcium channel blockers; *ASA,* acetyl salicylic acid.

**Table 2:** Biochemical, haematological, clinical and histological data of the study population

**Variable**	
Total cholesterol (mg/dL)	193.4 ± 38.5
HDL-cholesterol (mg/dL)	41.8 ± 9.3
LDL-cholesterol (mg/dL)	120.3 ± 32.9
Triglycerides (mg/dL)	156.2 ± 78.0
Apo A-1 (mg/dL)	122.5 ± 20.5
Apo B (mg/dL)	96.5 ± 23.3
Lp(a) (mg/dL)	37.4 ± 29.7
Glucose (mg/dL)	106.8 ± 33.9
CRP (mg/L)	2.6 ± 2.1
TNF-a (ng/dL)	1.5 ± 1.1
IL-6 (ng/dL)	3.7 ± 3.0
Homocysteine (μmol/L)	14.0 ± 4.7
ESR (mm/h)	21.7 ± 17.7
WBC (k/μL)	7.844 ± 1.899*10^3^
PLT (k/μL)	246.816 ± 64.314*10^3^
HCT (%)	39.8 ± 4.7
HbA1c (%)	6.0 ± 1.2
Fibrinogen (mg/dL)	401.3 ± 74.9
Antithrombin III (%)	82.6 ± 11.5
Protein S (%)	86.4 ± 16.0
Protein C (%)	102.1 ± 20.0
Right carotid artery stenosis (%)	55.6 ± 24.3
Left carotid artery stenosis (%)	84.3 ± 9.5
Clusterin expression on carotid tissue (density)	136.3 ± 18.0
Clusterin expression on carotid tissue (ratio per area) (%)	45.0 ± 18.8

Values are expressed as mean ± SD. *HDL*, high density lipoprotein; *LDL*, low density lipoprotein; *Apo A-1*, apolipoprotein A-1; *Apo B*, apolipoprotein B; *Lp(a),* lipoprotein (a); *CRP,* C-reactive protein; *TNF-a*, tumor necrosis factor-a; *IL-6,* interleukin-6; *ESR*, erythrocyte sedimentation rate; *WBC*, white blood cells; *PLT*, platelets; *HCT*, hematocrit; *HbA1c*, glycosylated haemoglobin.

The density of CLU expression on carotid tissue (arbitrary units) was significantly higher in smokers than in non-smokers (139.8 ± 13.7 *vs.* 127.4 ± 24.4, *p* = 0.041). However, it was not different between men and women (133.9 ± 19.3 *vs.* 144.0 ± 10.4), normal, overweight and obese subjects (140.2 ± 21.9, 129.0 ± 17.6 and 143.0 ± 9.0 respectively), symptomatic and asymptomatic patients (131.5 ± 21.3 *vs.* 141.1 ± 12.8). It was similar in hypertensives and normotensives (136.4 ± 16.7 *vs.* 135.5 ± 25.3), in diabetics and non-diabetics (135.9 ± 22.6 *vs.* 136.5 ± 16.1) as well as in patients suffering from CAD or not (141.7 ± 15.3 vs. 130.6 ± 17.4).

**Figure 1:**
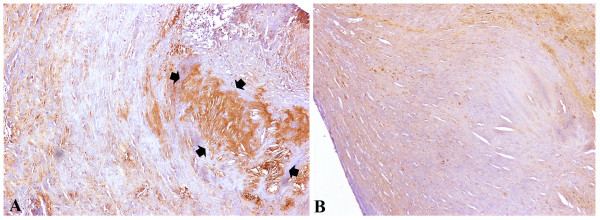
**Representative immunohistochemical figures, diaminobenzidine stain (brown colour, 100x original magnification).** Extensive and denser staining into the lipid core and macrophages of the atherosclerotic lesion in smokers (**A** - arrows) compared to non-smokers **(B)**.

Considering medication, density of CLU expression was significantly lower in patients on clopidogrel when compared to the rest not taking this pharmacological treatment (130.7 ± 22.0 *vs.* 142.3 ± 12.0, *p* = 0.045). Values of density were similar for the patients on the other treatments, which were examined. However, it is interesting to notice that there was a trend to lower density in patients receiving ASA (130.3 ± 23.1 *vs.* 141.5 ± 11.8, *p* = 0.055). Representative photomicrograhs of high and low CLU expression on carotid tissue are depicted on Figure 1 (A and B respectively).

Ratio of CLU expression per area of carotid tissue (%) did not differ between men and women (42.2 ± 19.7 *vs.* 53.8 ± 12.7), normal, overweight and obese subjects (40.9 ± 16.8, 46.9 ± 20.8 and 43.7 ± 18.8 respectively), symptomatic and asymptomatic patients (42.5 ± 19.9 *vs.* 47.5 ± 17.7). It was similar in smokers and non-smokers (45.3 ± 17.7 *vs.* 44.3 ± 22.2), hypertensives and normotensives (46.1 ± 19.2 *vs.* 39.2 ± 16.9), diabetics and non-diabetics (45.9 ± 17.8 *vs.* 44.6 ± 19.5) as well as in patients suffering from CAD or not (41.2 ± 17.5 *vs.* 46.7 ± 19.8). Considering medication, ratio per area did not exert any differences between patients on various pharmacological treatments. However, it was positively correlated with serum TNF-a concentration (*r* = 0.33, *p* = 0.040) but not with other measured parameters or with any pharmacological treatment.

Independent association between CLU density and CLU ratio per area of tissue with measured variables and medication was tested using linear regression analysis considering density or ratio per area as dependent variable. Clopidogrel intake was independent predictor of lower CLU density (*p* = 0.045) while TNF-a independent predictor of higher ratio per area in carotid tissue (*p* = 0.001).

## Discussion

Custerin/Apolipoprotein J is a secreted protein biosensor of oxidative stress, which is upregulated in a wide variety of pathological processes including aging, neurodegeneration, diabetes and atherosclerosis [8,9]. Although the precise function of the molecule is still under investigation, it has been accepted that CLU exerts cytoprotective and anti-inflammatory actions [11]. Additionally to its roles as secreted protein, it has been reported that might act intracellularly regulating homeostasis in human cells [14].

High serum concentration of CLU has been coupled to vascular damage and generalized stress conditions such as type II diabetes and coronary artery disease [8] and it has been associated with significant coronary stenosis [15].

In aortic tissue, CLU expression increases with atherosclerosis progression from fatty streaks to advanced lesions [7,10] while its presence in normal aortas is unobtrusive [7]. The molecule localization on vascular tissue could be attributed to HDL particles’ penetration from plasma, production through vascular smooth muscle cells (VSMC), release from activated platelets and retention in vascular wall through glycoproteins of extracellular matrix [16]. Recently, it has been shown that CLU expression in VSMC is induced by cellular RNA released from necrotic cells in atherosclerotic lesions through toll-like receptor 3 [11] while it seems that inhibits the apoptosis of VSMC *in vitro* by binding to modified-LDL particles [17]. The enhanced expression of CLU on injured tissue may serve the clearance of necrotic cell debris contributing to its protective role.

To our knowledge, a few studies enter into CLU and carotid atherosclerosis [12,13] while apolipoprotein’s expression on human carotid tissue and its association with parameters related to the disease development has not been examined. This was the purpose of the present work where the degree of CLU immunolocalization on carotid artery was tested for its relationship with demographic characteristics, blood parameters and pharmacological treatment of patients underwent internal carotid artery endarterectomy.

A number of parameters including classic biochemical and hematological parameters, thrombosis related parameters and inflammatory factors as well as the percentage of carotid stenosis were measured. Additionally, diabetic, hypertensive and patients with coronary artery disease participated in the study and pharmacological treatment was recorded aiming to find possible strong associations between CLU expression on carotid artery and parameters, which are related to atherosclerotic disease.

The results of the study showed that CLU expression density on carotid tissue was significantly higher in smoking subjects than in non-smoking ones. A large body of evidence confirms the relationship between smoke and atherosclerotic process. Framingham Heart Study conducted in 4255 men and women revealed that smoking is strongly associated with the risk of cerebral infarction [18]. Interestingly, the risk becomes significantly lower two years after smoking cessation while it returns to the risk for non-smokers five years after cessation. The prevalence of clinically significant internal carotid stenosis (≥50%) increases from the percentage of 4.4% in non-smokers to 7.3% in ex-smokers climbing to 9.5% in current smokers [19]. Smoking is thought to cause oxidative damage and is considered as a source of cellular stress. Since bibliographical data supports a role of CLU as a sensitive biomarker of oxidative stress [9] it is concluded that the higher expression of the molecule on the carotid tissue of smoking subjects reflects oxidative damage and cellular stress.

In the opposite site, molecule expression was significantly lower in patients on clopidogrel treatment and a trend for lower levels in patients taking ASA was observed. Both clopidogrel and ASA are anti-platelet agents that are indicated for the prevention of vascular ischemic events in patients, which are prone to atherosclerotic disease. The CAPRIE study, the bigger study until today where clopidogrel was used as antiplatelet agent, revealed that it is superb (75 mg/day) than ASA (325 mg/day) in secondary stroke prevention [20].

Since clopidogrel and ASA inhibit platelet aggregation in the endothelium impede atherosclerotic process exerting protective properties in vascular wall and this may explain the lower expression of CLU in carotid tissue. Another parameter, which might implicate in the lower presence of the molecule, is that CLU has been identified as one of the most abundant transcripts of human platelets and is highly expressed on extracellular membrane [21]. Under this point of view anti-platelet agents could inhibit a possible way of CLU localization on vascular tissue.

Another finding of the current study is that serum TNF-a concentration was significantly associated with CLU distribution on carotid artery of the patients and comprises an independent predictor of higher ratio CLU per area. Recent studies demonstrate the role of CLU on repressing NF-κB signaling [22,23] and it has been supported that interferes with TNF-a causing a negative regulation of inflammatory processes in the vessel wall [11]. The positive association between CLU distribution on carotid artery and serum TNF-a, which was observed in the present study reflects inflammation processes which are taking place during the development of atherosclerosis and possibly indicate the protective role of CLU in the vessel wall since it is known for its anti-proliferative, anti-apoptotic and anti-inflammatory properties [24]. CLU immunolocalization was not associated with the other inflammatory factors, which were measured. This result could be attributed to the small number of participants and the subsequent grate standard deviation, which was observed in these measurements. Additionally, pharmacological treatment influences inflammatory factors’ concentrations, i.e. statins therapy lowers CRP levels, as this was made clear in the JUPITER study [25].

CLU expression was not higher in diabetic patients or in patients suffering from coronary artery disease. Studies have demonstrated that serum CLU concentration is elevated in these patients’ categories [8,15] reflecting a generalized stress induction mechanism, which is related to these diseases. Certainly, it was supported that the elevated serum levels indicate vascular damage. A limitation of our study was that serum CLU concentration was not determined in order to reveal its possible association with the molecule immunolocalization on carotid tissue. However, carotid stenosis was not related with CLU tissue expression in our study.

CLU immunolocalization on carotid tissue was expressed as density and as ratio per area. Density refers to the qualitative evaluation of the stain (meaning how “strong” or how “weak” it is). Percentage refers to the positively stained area compared to the whole slide (expressed as % per area). However, further studies using reliable techniques could give explanations related to the different results concerning the association of density and ratio per area with each measured parameter.

Classical biochemical parameters and thrombosis related factors did not show any association with CLU immunolocalization on carotid artery.

## Conclusions

Concluding, the present study revealed that CLU, is highly expressed in carotid artery of smokers demonstrating cellular stress processes induced by oxidative stress and is positively correlated with serum TNF-a concentration reflecting inflammatory processes in vascular wall. The density of CLU was lower in the carotid artery of patients on anti-platelet treatment result, which is in accordance to the current knowledge about anti-platelet agents’ contribution to vascular protection.

## Methods

### Study population and protocol

The study was conducted in 42 patients (32 men and 10 women) suffering from high carotid stenosis (stenosis ≥ 70% to almost total occlusion) in Vascular Surgery Clinic of Red Cross Hospital, Athens, Greece. Subjects were Caucasians collected from the same geographic region. All patients met the criteria of carotid artery disease to be surgically operated, in accordance to the guidelines of the European Society of Vascular Surgery [26]. Carotid artery stenosis was diagnosed after digital subtraction angiography (DSA) of aortic arch, carotid and vertebral arteries and ultrasound angiography (colour duplex). Exclusion criteria were autoimmune or hematological disorders, heart or renal failure, liver dysfunction, chronic infection and malignancy. Anthropometric measurements (age, body weight, height, body mass index calculated as body weight (kg)/height^2^ (m^2^)) were performed and personal habits regarding smoking and physical activity were recorded. Smoking was premised as history of regular smoking (everyday use of tobacco for a long time as a habit). A detailed medical history questionnaire including previous myocardial infarction, carotid artery disease symptomatology, clinical syndromes such as coronary artery disease (CAD) and a positive family history of confirmed cardiovascular diseases considering first degree relatives was completed. Hypertension, when the patient was not taking any medication, was defined as systolic blood pressure ≥140 mmHg and/or diastolic blood pressure ≥90 mmHg and diabetes mellitus as two fasting plasma glucose levels of 126 mg/dL or higher.

Patient medication was recorded in detail and categorized as follows: beta adrenergic blockers (*β*-blockers), angiotensin converting enzyme (ACE) inhibitors, calcium channel blockers (CaCB), diuretics, clopidogrel, acetyl salicylic acid (ASA), antidiabetics, insulin and statins. Patients were taking their medication at least for one year.

Subjects underwent internal carotid artery endarterectomy using synthetic polytetrafluoroethylene (PTFE) patch (W.L.Gore & Associates, Newark, Delaware) under general anesthesia. Shunt for maintaining brain blood flow was not used in these operations. After endarterectomy, carotid plaque was carefully placed in 10% buffered formalin for further processing. Postoperatively, patients were normally recovered and left hospital without complications in about 4 days after surgery.

The ethical Committee of the Red Cross Hospital of Athens approved the study protocols and all patients gave written informed consent.

### Blood collection and analyses

Venous blood was collected at 8 p.m. the previous night of surgical operation after 6 hrs fast. Specimens for serum isolation were left to clot for 30 min at room temperature and were then centrifuged at 3000 rpm for 15 min at 4°C. Isolated samples were stored in aliquots at −80°C until analysis. For hematological determinations, EDTA tubes were used and measurements were performed immediately.

White blood cells (WBC), platelets (PLT), hematocrit (HCT), and glycosylated hemoglobin (HbA1c) were automatically measured in hematological analyzer (Beckman Coulter LH 750). Thrombosis related parameters as fibrinogen, antithrombin III, protein C and protein S were measured in Stago STA compact coagulation analyzer.

Serum glucose, total cholesterol, low density lipoprotein (LDL)-cholesterol, HDL-cholesterol, triacylglycerols, apolipoproteins A-1 and B, lipoprotein (a) (Lp(a)) and homocysteine were determined in Cobas Integra 800 (Roche) biochemical analyzer.

Sensitive ELISA sandwich immunoassays (Immunodiagnostik AG, Bensheim, Germany) were used for the determination of tumor necrosis factor-a (TNF-a), interleukin-6 (IL-6) and C-reactive protein (high sensitive CRP) according to manufacturer’s instructions. Erythrocyte sedimentation rate (ESR) was estimated after 1 hr in EDTA anti-coagulated blood.

### Immunohistochemistry

Representative serial sections for immunohistochemistry were cut at 3 μm from paraffin-embedded specimens, mounted on poly-L-lysine slides, and allowed to dry at 37°C overnight. Before applying the primary antibody, sections were treated in a microwave oven at 750 W for 3 cycles of 5 min each, in 10 mmol/L sodium citrate buffer pH 6.0. Slides were left to cool down in the antigen retrieval solution for 20 minutes. Endogenous peroxidase activity was quenched by first incubating the specimens for 5 min in 3% hydrogen peroxide (supplied by user). The specimens were then incubated with the primary antibody against Apo-J (clone sc-6420, goat polyclonal antibody, Santa Cruz Biotechnology Inc, CA) in a dilution 1:200, followed by sequential incubations with biotinylated link antibody and peroxidase-labelled streptavidin (LSAB + System-HRP, Code K0690, Dako, Glostrup, Denmark). Staining was completed after incubation with substrate-chromogen solution (Large Volume DAB+, code K3468, Dako, Glostrup, Denmark). Hematoxylin was used for counterstaining. Known positive controls, as well as negative controls (sections in which the primary antibody was substituted with non-immune mouse serum) were also stained in each run.

### Image analysis

Images of the immunohistochemically stained sections were captured with a Nikon DS-2 MW colour CCD digital camera mounted on a Nikon Eclipse 80i microscope (Nikon Co, Tokyo, Japan) under 20x original magnification and stored as high quality JPG files. Three to five representative images per section were captured. Images were then analyzed with Image-Pro Plus 5.1 software (Media Cybernetics, SilverSpring, MD). The parameters measured by the image analysis program were the intensity of CLU staining in the atherosclerotic plaque and the extent of staining as well in relation to the total area [27]. Brown diaminobenzidine (DAB) staining, indicative of CLU expression, was distinguished from the blue hematoxylin counterstain with hue thresholds. Color threshold settings of DAB-stained pixels were set manually prior to analysis and left unchanged throughout. Total atherosclerotic areas in each image were circumscribed using the “area of interest” tool included in the Image-Pro Plus program in combination with an Intuos digitizing pen (Wacom Technology; Vancouver, WA), and the total circumscribed area was calculated. The area stained by the antibody of interest was identified and calculated using the software colour cube algorithm. The staining intensity levels of CLU were measured using on a linear scale ranging from 0 (nondetectable) to 255 (highest intensity). The pathologist performing the computerized image analysis was blinded to the clinical outcome of the patients.

### Statistical analysis

Data were analyzed using SPSS software 17.0. Statistical significance was accepted at *p* < 0.05. Values were expressed as mean ± SD for continuous variables and as percentage for categorical data. Student’s *t*-test was performed to compare continuous variables between two groups and analysis of variance (ANOVA) for more than two groups. Chi-square test was used to evaluate differences between categorical data. Correlations between continuous variables were indicated by Pearson correlation coefficient (r). Linear regression analysis was performed to denote independent associations with CLU expression on carotid tissue.

## Abbreviations

ASA: Acetyl salicylic acid; ACE: Angiotensin converting enzyme; Apo A-1, Apo B: Apolipoprotein A-1, B; β-blockers: Beta adrenergic blockers; BMI: Body mass index; CaCB: Calcium channel blockers; CETP: Cholesteryl ester transfer protein; CLU: Clusterin; CAD: Coronary artery disease; DAB: Diaminobenzidine; DSA: Digital subtraction angiography; ESR: Erythrocyte sedimentation rate; HbA1c: Glycosylated hemoglobin; HCT: Hematocrit; HDL: High density lipoprotein; hsCRP: High sensitive C-reactive protein; IL-6: Intereleukin-6; Lp(a): Lipoprotein (a); LDL: Low density lipoprotein; PLT: Platelets; PTFE: Polytetrafluoroethylene; TNF-a: Tumor necrosis factor-a; VSMC: Vascular smooth muscle cells; WBC: White blood cells.

## Competing interests

The authors declare that they have no competing interests.

## Authors’ contributions

AY participated in analysis and interpretation of data and involved in drafting the manuscript. GA carried out immunohistological examinations, performed the image analysis and helped to draft the manuscript. CG participated in the design of the study, the acquisition, analysis and interpretation of data. DP conceived of the study, participated in its design and coordination. All authors read and approved the final manuscript.
